# Pudendal Nerve Entrapment Within Alcock’s Canal as a Compartment-Like Syndrome: A Systematic Review

**DOI:** 10.7759/cureus.112846

**Published:** 2026-07-17

**Authors:** Jideofor Okoye, Moazer Ibrahim Hamid Mohammed, Divyank Subhedar, Mohamed Ishaq Abdalla Mohamed, Shashwat Shetty, Rizwan Ali

**Affiliations:** 1 Trauma and Orthopaedics, Mersey and West Lancashire Teaching Hospital, NHS Foundation Trust, England, GBR; 2 Surgery, British United Provident Association, Jeddah, SAU; 3 General Practice, Barking, Havering and Redbridge University Hospitals NHS Trust, London, GBR; 4 Orthopaedics, Hillingdon Hospital, Uxbridge, GBR; 5 Medicine, Jinnah Sindh Medical College, Karachi, PAK

**Keywords:** alcock’s canal, chronic pelvic pain, compartment syndrome, pudendal nerve entrapment, pudendal neuralgia

## Abstract

Pudendal nerve entrapment (PNE) within Alcock’s canal is an under-recognized cause of chronic pelvic and perineal pain, increasingly conceptualized as a compartment-like syndrome due to sustained neurovascular compression within a confined fascial space. This systematic review evaluates the anatomical, pathophysiological, diagnostic, and therapeutic evidence supporting this hypothesis. A comprehensive literature search was conducted across PubMed, Embase, Scopus, and the Cochrane Library from April 2026 to May 1, 2026, in accordance with Preferred Reporting Items for Systematic Reviews and Meta-Analyses (PRISMA) guidelines. Ten studies were included in the final synthesis, comprising observational studies, cadaveric anatomical analyses, imaging-based diagnostic studies, and interventional and surgical outcome studies. Sample sizes ranged from a cadaveric series of 15-20 specimens to clinical cohorts exceeding 200 patients. Evidence consistently demonstrated that repetitive mechanical stress, fascial constriction, and impaired venous drainage within Alcock’s canal may lead to ischemic neuropathy and progressive pudendal dysfunction. Diagnostic modalities such as magnetic resonance (MR) neurography, Nantes criteria, and nerve blocks improved clinical identification, while surgical decompression showed variable but often favorable outcomes. However, significant heterogeneity, small sample sizes, and a lack of standardized outcome measures limit definitive conclusions. Overall, current evidence supports a compartment-like pathophysiology, although further prospective validation is required.

## Introduction and background

Chronic pelvic pain affects approximately 15%-25% of women worldwide and represents a major source of disability, healthcare utilization, and impaired quality of life [1]. Among the neuropathic causes of chronic pelvic pain, pudendal neuralgia has emerged as an increasingly recognized but frequently underdiagnosed disorder. Pudendal neuralgia originates from irritation, inflammation, or entrapment of the pudendal nerve, a mixed sensory, motor, and autonomic nerve arising from the S2-S4 sacral roots [2]. Patients commonly present with burning perineal pain, dyspareunia, urinary dysfunction, anorectal symptoms, and pain exacerbated by sitting. Diagnostic delays are common, with many patients undergoing years of multidisciplinary evaluation before definitive recognition of pudendal nerve pathology. The pudendal nerve traverses several anatomically vulnerable regions during its pelvic course, most notably between the sacrospinous and sacrotuberous ligaments and within Alcock’s canal [3]. Alcock’s canal is a fibro-fascial tunnel formed by the obturator internus fascia along the lateral wall of the ischioanal fossa [4]. Anatomical and surgical studies have demonstrated that this confined space predisposes the pudendal neurovascular bundle to chronic compression, repetitive traction injury, and vascular compromise. Reported etiologies include prolonged sitting, cycling, pelvic surgery, childbirth trauma, pelvic floor hypertonicity, and chronic constipation. Similar to peripheral entrapment neuropathies such as carpal tunnel syndrome, prolonged pudendal nerve compression may impair intraneural microcirculation, resulting in venous congestion, neural edema, ischemia, demyelination, and progressive neuropathic dysfunction [5].

Increasing evidence suggests that pudendal nerve entrapment (PNE) within Alcock’s canal may behave physiologically as a chronic compartment-like syndrome [6]. Chronic neurovascular compression within a restricted fascial compartment resembles the pathophysiological cascade observed in chronic compartment syndromes, where elevated tissue pressure compromises neural perfusion and axonal transport. Imaging studies using magnetic resonance (MR) neurography have demonstrated pudendal nerve edema, distortion, and signal abnormalities at common entrapment sites, while surgical decompression studies have reported symptomatic improvement rates ranging from 60% to 80% in carefully selected patients [7]. Despite growing recognition, no universally accepted diagnostic gold standard currently exists. The Nantes criteria remain the most widely adopted clinical framework and include five essential features: pain within the pudendal territory, worsening with sitting, absence of nocturnal awakening, no objective sensory deficit, and positive response to pudendal nerve block [8]. The primary aim of this systematic review is to evaluate the current evidence regarding PNE within Alcock’s canal as a potential compartment-like syndrome characterized by chronic neurovascular compression within a confined anatomical space. Secondary objectives include assessing the anatomical and pathophysiological mechanisms underlying pudendal neuropathy, reviewing available diagnostic and imaging modalities, evaluating conservative and surgical treatment strategies, and analyzing functional as well as long-term clinical outcomes reported in the literature.

## Review

Materials and methods

Search Strategy

This systematic review was conducted in accordance with the Preferred Reporting Items for Systematic Reviews and Meta-Analyses (PRISMA) 2020 guidelines to ensure methodological rigor, transparency, and reproducibility throughout the review process [9]. A comprehensive and systematic electronic literature search was undertaken to identify all relevant studies investigating PNE within Alcock’s canal and exploring its potential pathophysiological similarities to compartment syndrome. Four major electronic databases were searched: PubMed/MEDLINE, Embase, Scopus, and the Cochrane Library. The search encompassed all studies published from January 1987 to May 2026, reflecting the period during which significant literature on pudendal neuralgia, PNE, and surgical decompression techniques became available. The literature search was conducted between April 15, 2026, and May 1, 2026. A structured search strategy was developed with input from the review team and incorporated both free-text keywords and controlled vocabulary terms, including Medical Subject Headings (MeSH) for PubMed and Emtree terms for Embase, where applicable. The search strategy was adapted appropriately for each database to maximize sensitivity while maintaining specificity. Keywords and subject headings were combined using Boolean operators (“AND” and “OR”) to capture all potentially relevant studies.

The principal search terms included “pudendal nerve entrapment,” “pudendal neuralgia,” “Alcock canal,” “Alcock’s canal syndrome,” “pudendal canal syndrome,” “compartment syndrome,” “pelvic neuropathy,” “neurovascular compression,” “pudendal nerve compression,” “pelvic pain syndrome,” and “pudendal decompression.” Various synonyms, spelling variations, and related concepts were also incorporated where appropriate to broaden the search and reduce the likelihood of missing relevant studies. In addition to electronic database searching, a manual review of the reference lists of all included studies, review articles, and relevant clinical guidelines was undertaken to identify additional publications that may not have been captured through the primary search strategy. Citation tracking and backward reference searching were also performed when applicable. Gray literature sources, conference proceedings, and unpublished studies were considered if sufficient methodological and outcome data were available. All retrieved records were exported into a reference management software package, where duplicate citations were identified and removed prior to screening. Following deduplication, titles and abstracts were screened for relevance, and full-text articles were subsequently assessed against predefined eligibility criteria. The search process and study selection workflow were documented using a PRISMA 2020 flow diagram to ensure transparency and reproducibility of the review methodology.

Eligibility Criteria

Study eligibility was determined using the Population, Intervention/Exposure, Comparator, and Outcomes (PICO) framework [10]. The study population (P) included adult human subjects diagnosed with PNE, pudendal neuralgia, or compression neuropathy involving Alcock’s canal. The intervention/exposure (I) comprised anatomical compression, neurovascular entrapment, diagnostic evaluation, conservative management, image-guided interventions, or surgical decompression of the pudendal nerve. Comparators (C), where available, included asymptomatic controls, patients without pudendal neuropathy, alternative treatment modalities, or preoperative versus postoperative cohorts. The outcomes (O) of interest included anatomical findings, pathophysiological mechanisms, diagnostic accuracy, pain reduction, functional improvement, surgical outcomes, recurrence rates, and long-term neurological recovery. Inclusion criteria consisted of human studies involving anatomical, diagnostic, pathophysiological, or therapeutic evaluation of PNE within Alcock’s canal. Eligible study designs included randomized controlled trials, cohort studies, case-control studies, observational studies, cross-sectional studies, and case series published in the English language. Exclusion criteria included animal studies, isolated case reports, editorials, expert opinions without primary patient data, conference abstracts lacking complete manuscripts, and non-English publications.

Study Selection

The selection of studies followed a stepwise process consistent with PRISMA recommendations. All search results were initially compiled, and duplicates were removed. The remaining records were screened by reviewing titles and abstracts to identify studies that were potentially relevant to the research question. Full-text versions of these studies were then assessed in more detail against the eligibility criteria. Studies that did not meet the criteria were excluded at this stage, and reasons for exclusion were noted to maintain transparency. The process was documented using a PRISMA flow diagram.

Data Extraction

Data extraction was independently performed by two reviewers using a standardized data collection form developed prior to study analysis. Extracted variables included author names, year of publication, country of origin, study design, sample size, population characteristics, diagnostic criteria utilized, anatomical findings, proposed pathophysiological mechanisms, imaging modalities, electrophysiological assessments, conservative or surgical therapeutic interventions, functional outcomes, postoperative outcomes, recurrence rates, and long-term follow-up findings. Particular emphasis was placed on evidence supporting neurovascular compression, ischemic neuropathy, fascial compartment restriction, and anatomical entrapment patterns within Alcock’s canal.

Risk of Bias Assessment

Methodological quality and risk of bias were independently assessed according to study design using validated critical appraisal tools. Randomized controlled trials were evaluated using the Cochrane Risk of Bias Tool version 2 (RoB 2) [11]. Cohort studies and observational studies were assessed using the Newcastle-Ottawa Scale (NOS) [12], while case series were evaluated using the Joanna Briggs Institute (JBI) critical appraisal checklist [13]. Diagnostic accuracy studies involving imaging modalities or electrophysiological investigations were assessed using the QUADAS-2 tool [14]. Studies were categorized as low, moderate, or high risk of bias based on methodological quality, participant selection, outcome assessment, follow-up adequacy, and reporting transparency. Overall risk of bias ratings were derived from the individual domain assessments recommended by each appraisal tool. Studies were classified as low risk when the majority of domains demonstrated low risk without major methodological concerns, moderate risk when one or more domains raised some concerns that were unlikely to substantially alter the overall findings, and high risk when multiple domains demonstrated significant methodological limitations or high risk of bias that could meaningfully affect study validity.

Data Synthesis

Due to significant heterogeneity among included studies regarding study design, patient populations, diagnostic methodologies, anatomical assessments, intervention strategies, and reported outcome measures, quantitative meta-analysis was not feasible. Therefore, a qualitative narrative synthesis was performed. Studies were grouped according to thematic categories including anatomical investigations, diagnostic studies, imaging evaluations, interventional procedures, surgical decompression outcomes, and long-term functional recovery. Quantitative meta-analysis was not considered appropriate because of substantial clinical and methodological heterogeneity across the included studies, including differences in study design, patient populations, diagnostic criteria (clinical, imaging, and neurophysiological), intervention types (conservative management, image-guided procedures, and surgical decompression), outcome measures, and duration of follow-up. Consequently, a qualitative narrative synthesis was undertaken.

Registration

This systematic review was conducted in accordance with the PRISMA 2020 guidelines. A review protocol was developed before study selection and data extraction to ensure methodological consistency; however, the protocol was not registered in PROSPERO. All review methods were predefined and applied throughout the study to enhance transparency and reproducibility.

Results

Study Selection Process

**Figure 1:**
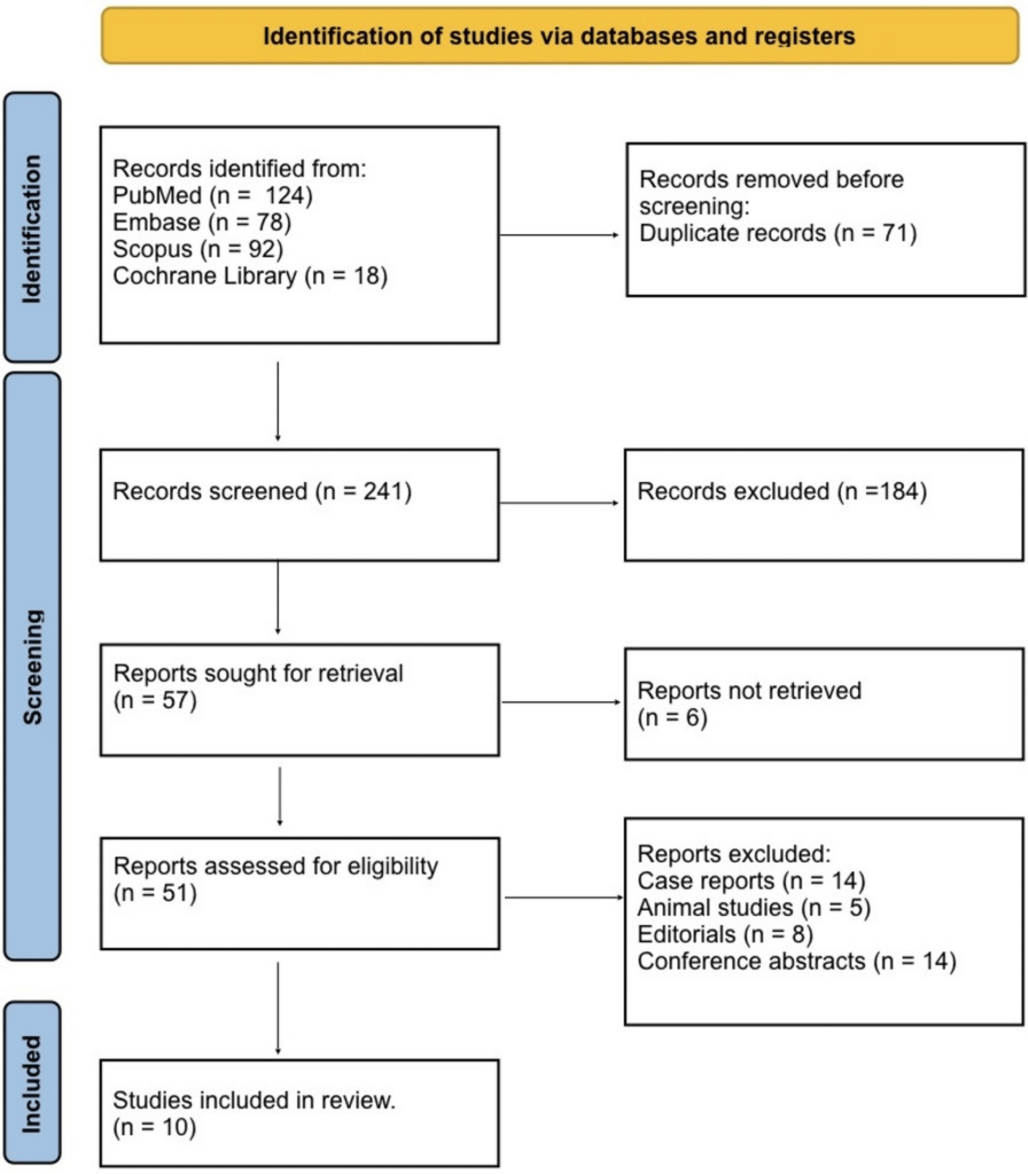
PRISMA 2020 flow diagram PRISMA: Preferred Reporting Items for Systematic Reviews and Meta-Analyses

Figure 1 shows a total of 312 records across four electronic databases, including 124 records from PubMed, 78 from Embase, 92 from Scopus, and 18 from the Cochrane Library. Following the removal of 71 duplicate records, 241 studies remained for title and abstract screening. During the screening phase, 184 records were excluded due to irrelevance to PNE, lack of focus on Alcock’s canal, absence of primary clinical data, or failure to meet predefined eligibility criteria. Subsequently, 57 reports were sought for full-text retrieval, of which six articles could not be accessed despite institutional and manual search efforts. A total of 51 full-text articles were assessed for eligibility. Among these, 14 studies were excluded because they were isolated case reports, five were excluded due to animal-based methodology, eight were excluded as editorials or expert opinion articles lacking primary patient data, and 14 additional studies were excluded because they consisted of conference abstracts, duplicate datasets, or narrative reviews or lacked sufficient methodological detail for inclusion. Ultimately, 10 studies met the final inclusion criteria and were incorporated into the qualitative synthesis of this systematic review.

Characteristics of the Selected Studies

Table 1 shows that the included studies comprised observational clinical studies, anatomical cadaveric analyses, imaging-based diagnostic studies, and interventional trials evaluating PNE within Alcock’s canal, with sample sizes ranging from 15 cadavers to over 200 patients. Early studies by Amarenco et al. first characterized pudendal canal syndrome as a chronic neuropathy resulting from compression within Alcock’s canal, leading to ischemic injury, sensory dysfunction, and urinary symptoms [15]. Robert et al. later identified key anatomical entrapment zones between the sacrospinous and sacrotuberous ligaments and within Alcock’s canal, demonstrating that repetitive mechanical compression and fibrosis contribute significantly to neuropathic pain development [16]. Beco et al. further reported that surgical decompression improved pain, continence, and sexual dysfunction in patients with refractory pudendal neuralgia, while also highlighting abnormal pudendal nerve terminal motor latency associated with chronic compression [17]. Diagnostic standardization was significantly improved by Labat et al. through validation of the Nantes criteria, which remain the principal diagnostic framework for pudendal neuralgia despite overlap with other chronic pelvic pain disorders [18]. Imaging advances by Filler demonstrated the value of MR neurography in visualizing nerve edema, distortion, and enlargement within Alcock’s canal, thereby improving localization of entrapment and surgical planning [19]. Subsequent studies focused on recurrent disease, autonomic dysfunction, and image-guided therapeutic interventions. Hibner et al. reported that repeat decompression surgery could provide partial-to-significant pain improvement in selected patients with recurrent PNE, although outcomes were poorer in longstanding neuropathy with fibrosis [20]. Possover and Forman additionally emphasized the autonomic component of PNE, linking chronic compression with urinary and pelvic floor dysfunction through involvement of pelvic autonomic pathways [21]. In a randomized controlled trial, Labat et al. demonstrated that computed tomography (CT)-guided pudendal nerve infiltration with corticosteroids provided temporary pain relief but no significant long-term advantage over local anesthetic alone, suggesting that structural compression remains a major pathogenic factor [22]. Anatomical validation studies by Bendtsen et al. and Soucy et al. showed that ultrasound-guided Alcock's canal blockade improves procedural precision and anatomical targeting compared with traditional techniques, supporting minimally invasive approaches for both diagnosis and treatment [23,24]. Overall, the literature demonstrates that PNE is a multifactorial neuropathic condition involving chronic compression, fibrosis, ischemia, and autonomic dysfunction, with advances in diagnostic criteria, imaging, and targeted interventions significantly improving clinical management despite persistent challenges in delayed or recurrent cases.

**Table 1:** Characteristics of selected studies NR: not reported; MR: magnetic resonance; CT: computed tomography; PNTML: pudendal nerve terminal motor latency

Authors & year	Study design	Sample size (n)	Population (P)	Exposure/condition (I)	Comparator (C)	Diagnostic criteria used	Outcomes (O)	Pathophysiological findings	Anatomical impact	Diagnostic/surgical techniques	Functional outcomes	Long-term outcomes	Complications/adverse events	Key conclusion
Amarenco et al., 1987 [15]	Observational clinical study	NR	Patients with chronic perineal pain and urinary dysfunction	Pudendal nerve compression within Alcock’s canal	No formal comparator	Clinical and neurophysiological assessment	Characterization of pudendal canal syndrome	Chronic neural compression causing ischemic neuropathy and sensory dysfunction	Compression beneath obturator internus fascia within Alcock’s canal	Electrophysiology, pudendal nerve assessment	Symptomatic improvement after targeted interventions	Established chronicity of pudendal neuropathy	Persistent symptoms in delayed diagnosis	First detailed description of pudendal canal syndrome
Robert et al., 1998 [16]	Anatomical cadaveric and clinical correlation study	NR	Patients with chronic perineal pain	Pudendal nerve entrapment between sacrospinous/sacrotuberous ligaments and Alcock’s canal	Cadaveric anatomical controls	Anatomical and surgical exploration	Identification of entrapment zones	Repetitive compression and fibrosis causing neuropathy	Entrapment at interligamentous space and Alcock’s canal	Cadaveric dissection and decompression surgery	Reduction in pain after decompression	Durable relief in selected patients	Surgical morbidity not extensively reported	Established anatomical basis for pudendal nerve entrapment
Beco et al., 2004 [17]	Prospective case series	74	Patients with pudendal neuralgia and pelvic dysfunction	Pudendal canal syndrome treated surgically	Preoperative status comparison	Clinical examination, PNTML testing	Pain relief and continence improvement	Chronic compression associated with motor latency abnormalities	Fascial restriction within pudendal canal	Bilateral pudendal nerve decompression	Improvement in pain, continence, and sexual symptoms	Sustained benefit in majority of cases	Wound complications and incomplete pain relief in some patients	Surgical decompression may improve refractory pudendal neuralgia
Labat et al., 2008 [18]	Diagnostic criteria validation study	135	Patients with suspected pudendal neuralgia	Pudendal nerve entrapment	Chronic pelvic pain patients without pudendal neuralgia	Nantes criteria	Diagnostic specificity and reproducibility	Mechanical irritation and chronic neural inflammation	Entrapment along Alcock’s canal and ligamentous structures	Nantes criteria and diagnostic nerve blocks	Improved diagnostic standardization	Enabled long-term comparative assessment	Diagnostic overlap with other pelvic pain syndromes	Nantes criteria remain the diagnostic standard
Filler, 2009 [19]	Imaging-based observational study	NR	Patients with refractory pudendal neuralgia	Pudendal nerve entrapment evaluated with MR neurography	Conventional imaging	MR neurography findings	Diagnostic localization of entrapment	Nerve edema and chronic compressive injury visualized on imaging	Enlargement/distortion of pudendal nerve at Alcock’s canal	MR neurography and image-guided intervention	Improved localization for surgery	Better targeted decompression strategies	Limited imaging availability and cost	MR neurography improved detection of pudendal neuropathy
Hibner et al., 2010 [20]	Retrospective surgical review	19	Patients with persistent pudendal neuralgia after prior surgery	Recurrent pudendal nerve entrapment	Primary decompression cohort	Clinical examination and prior operative history	Outcomes after repeat decompression	Persistent fibrosis and recurrent neural compression	Re-entrapment around decompressed nerve segments	Repeat transgluteal neurolysis	Partial-to-significant pain improvement	Less favorable outcomes in longstanding disease	Persistent neuropathic pain and scar-related morbidity	Reoperative decompression can benefit selected patients
Possover and Forman, 2014 [21]	Clinical observational study	NR	Patients with pudendal neuralgia and voiding dysfunction	Alcock's canal syndrome with autonomic involvement	Patients without neurogenic dysfunction	Clinical examination and nerve palpation	Correlation between entrapment and urinary symptoms	Chronic compression contributing to pelvic autonomic dysfunction	Pudendal nerve involvement extending into pelvic floor pathways	Selective nerve blockade and targeted therapy	Improvement in urinary and pelvic pain symptoms	Chronic neurogenic sequelae noted in delayed cases	Incomplete symptom resolution in severe neuropathy	Pudendal entrapment may contribute to autonomic pelvic dysfunction
Labat et al., 2017 [22]	Randomized controlled trial	201	Patients with pudendal neuralgia	CT-guided pudendal nerve infiltration with corticosteroid	Local anesthetic alone	Nantes criteria and imaging-guided diagnosis	Pain improvement after infiltration	Inflammatory and compressive mechanisms implicated	Compression near sacrospinous ligament and Alcock’s canal	CT-guided pudendal nerve infiltration	Temporary pain improvement in some patients	No long-term superiority with corticosteroids	Injection discomfort and transient numbness	Steroid addition did not significantly improve long-term outcomes
Bendtsen et al., 2016 [23]	Cadaveric anatomical validation study	20 hemipelves	Cadaveric specimens for pudendal nerve block assessment	Ultrasound-guided Alcock's canal blockade	Traditional ischial spine approach	Ultrasound anatomical localization	Feasibility and precision of blockade	Improved targeting reduces incomplete blockade risk	Sonographic visualization of pudendal nerve in Alcock’s canal	Ultrasound-guided pudendal block	Enhanced procedural precision	Potential application in longitudinal management	Operator dependency	Ultrasound guidance improves procedural anatomical accuracy
Soucy et al., 2020 [24]	Cadaveric comparative study	15 cadavers	Cadaveric evaluation of pudendal blockade techniques	Alcock's canal versus ischial spine injection sites	Comparative injection site analysis	Ultrasound-guided injection mapping	Accuracy of pudendal nerve targeting	Demonstrated confined neurovascular relationships within canal	Confirmed accessibility of pudendal nerve within Alcock’s canal	Ultrasound-guided cadaveric dye injection	Improved understanding of procedural anatomy	Supports minimally invasive approaches	Cadaveric limitations without clinical outcomes	Alcock's canal targeting may improve blockade precision

Risk of Bias Assessment

Table 2 shows that the included studies demonstrated variable methodological quality depending on study design, with risk of bias tools applied accordingly. Amarenco et al. showed moderate to high risk of bias due to small sample size, lack of comparator group, and non-standardized outcome reporting [15]. Robert et al. had moderate risk given cadaveric variability and limited clinical correlation [16]. Beco et al. demonstrated moderate risk due to the absence of a control group and subjective outcome measures [17]. Labat et al. had low to moderate risk as the Nantes criteria improved diagnostic standardization, although some incorporation bias remained [18]. Imaging-based evidence from Filler showed moderate risk due to referral and interpretation bias [19]. Surgical outcome studies such as Hibner et al. and Possover and Forman carried moderate to high and moderate risk, respectively, due to retrospective design and small heterogeneous cohorts [20,21]. The randomized controlled trial by Labat et al. demonstrated low risk of bias overall despite limited long-term follow-up [22]. Cadaveric validation studies by Bendtsen et al. and Soucy et al. showed low to moderate, primarily due to lack of in vivo confirmation and limited sample sizes [23,24].

**Table 2:** Risk of bias assessment JBI: Joanna Briggs Institute; QUADAS-2: Quality Assessment of Diagnostic Accuracy Studies-2; NOS: Newcastle-Ottawa Scale; RoB 2: Cochrane Risk of Bias Tool version 2; MR neurography: magnetic resonance neurography

Study	Study design	Risk of bias tool used	Risk of bias rating	Major sources of bias/limitations	Justification
Amarenco et al., 1987 [15]	Observational clinical study	JBI Checklist for Case Series	Moderate-high risk	Small sample size, absence of comparator group, non-standardized outcomes, retrospective symptom assessment	Early exploratory study establishing pudendal canal syndrome but lacking validated diagnostic criteria and long-term standardized follow-up
Robert et al., 1998 [16]	Anatomical cadaveric and clinical correlation study	Anatomical Study Quality Appraisal Framework/JBI	Moderate risk	Limited clinical correlation, cadaveric variability, selection bias in surgical cohort	Strong anatomical detail but limited prospective clinical outcome data
Beco et al., 2004 [17]	Prospective case series	JBI Checklist for Case Series	Moderate risk	No randomization, no control group, subjective pain reporting, potential surgeon-related bias	Prospective methodology improved reliability, but lack of comparator limits causal inference
Labat et al., 2008 [18]	Diagnostic validation study	QUADAS-2	Low-moderate risk	Diagnostic incorporation bias, absence of definitive gold standard	Widely accepted Nantes criteria improved standardization, though diagnostic overlap with pelvic pain syndromes persists
Filler, 2009 [19]	Imaging observational study	NOS	Moderate risk	Referral bias, imaging interpretation variability, limited external validation	MR neurography demonstrated diagnostic utility, but reproducibility and accessibility remained limited
Hibner et al., 2010 [20]	Retrospective surgical review	NOS	Moderate-high risk	Retrospective design, small cohort, selection bias, lack of blinding	Reoperative surgical outcomes valuable but limited by heterogeneous patient population
Possover and Forman, 2014 [21]	Clinical observational study	NOS	Moderate risk	Small sample, subjective symptom assessment, limited standardized follow-up	Demonstrated autonomic implications of pudendal entrapment but lacked controlled comparison
Labat et al., 2017 [22]	Randomized controlled trial	RoB 2	Low risk	Limited long-term follow-up, procedural variability	Strongest methodological quality among included studies with randomized comparative design
Bendtsen et al., 2016 [23]	Cadaveric anatomical validation study	Anatomical Study Quality Appraisal Framework	Low-moderate risk	Cadaveric tissue differences from living anatomy, no clinical outcomes	High anatomical precision and procedural reproducibility despite lack of patient-based outcomes
Soucy et al., 2020 [24]	Cadaveric comparative study	Anatomical Study Quality Appraisal Framework	Moderate risk	Limited sample size, absence of clinical validation	Useful procedural anatomical data but findings require in vivo confirmation

Discussion

The findings from the included studies collectively support the concept that PNE within Alcock’s canal may behave as a compartment-like syndrome rather than a simple static entrapment neuropathy. Alcock’s canal is anatomically formed by the fascia of the obturator internus muscle, creating a relatively rigid fibroosseous tunnel through which the pudendal neurovascular bundle passes. Similar to compartment syndromes seen in the extremities, this confined anatomical environment may predispose the nerve to pressure-related ischemia, impaired venous drainage, and chronic neural compression. Amarenco et al. first described pudendal canal syndrome as a chronic neuropathic disorder characterized by perineal pain and urinary dysfunction resulting from prolonged compression within Alcock’s canal, suggesting an ischemic mechanism of neural injury [15]. This theory is strengthened by the observation that symptoms are often aggravated by sitting, a position known to increase pelvic floor pressure and potentially elevate pressure within the canal itself. Robert et al. further supported this concept through anatomical studies identifying fixed compression points between the sacrospinous and sacrotuberous ligaments and within Alcock’s canal, where repetitive mechanical stress and fibrosis may progressively increase pressure around the nerve [16]. These anatomical findings parallel the pathophysiology of chronic compartment syndromes in which restricted fascial boundaries prevent adequate pressure dissipation, ultimately compromising neural perfusion.

Several studies within the table provide indirect supportive evidence of this compartment-like physiology. Beco et al. demonstrated abnormal pudendal nerve terminal motor latency in patients with pudendal neuralgia, indicating chronic functional impairment likely secondary to prolonged neural compression and ischemia [17]. The improvement in pain, continence, and sexual dysfunction following surgical decompression further supports the idea that relieving pressure within the canal restores neural function, similar to decompressive fasciotomy in compartment syndromes. Imaging studies by Filler also strengthen this hypothesis by demonstrating nerve edema, enlargement, and signal abnormalities on MR neurography consistent with chronic compressive neuropathy [19]. Such radiological findings resemble imaging changes observed in nerves exposed to prolonged compartmental pressure elsewhere in the body. Moreover, Possover and Forman highlighted that chronic pudendal compression may also affect autonomic pelvic pathways, producing urinary and pelvic floor dysfunction, which suggests that sustained pressure within Alcock’s canal may compromise not only somatic sensory fibers but also autonomic neural components [21]. The recurrent fibrosis and re-entrapment described by Hibner et al. after prior decompression surgery additionally support the concept of an ongoing pathological pressure environment within the canal [20].

The procedural and anatomical studies included in the review further reinforce the compartment-like nature of PNE. Bendtsen et al. and Soucy et al. demonstrated that ultrasound-guided blockade specifically targeting Alcock’s canal allows more accurate localization of the pudendal nerve compared with traditional ischial spine approaches, confirming the confined and anatomically consistent relationship between the nerve and surrounding fascial structures [23,24]. The effectiveness of image-guided decompression or infiltration procedures suggests that pathology is highly localized within this restricted anatomical space. Furthermore, the temporary symptomatic improvement observed after CT-guided pudendal nerve infiltration in the randomized trial by Labat et al. may reflect the transient reduction of local inflammation and pressure around the nerve, although the lack of long-term benefit indicates that persistent structural compression remains the primary pathological mechanism [22]. Collectively, these findings justify the theory that Alcock’s canal functions similarly to a closed fascial compartment in which chronic pressure elevation contributes to ischemia, fibrosis, neural dysfunction, and progressive neuropathy. Although a formal GRADE assessment was not performed because of the substantial heterogeneity of study designs and outcomes, the overall certainty of the available evidence is considered low to moderate. This reflects the predominance of observational studies, anatomical investigations, and relatively small clinical cohorts despite generally consistent findings across multiple study types.

Despite the growing evidence supporting this theory, the current literature remains limited by the absence of direct intracompartmental pressure measurements within Alcock’s canal. Most studies provide indirect evidence based on anatomy, imaging, neurophysiology, and response to decompression rather than direct physiological confirmation. Nevertheless, the consistent demonstration of fixed entrapment zones, pressure-related symptom exacerbation, imaging evidence of nerve edema, electrophysiological abnormalities, and clinical improvement following decompression collectively provide strong support for considering PNE within Alcock’s canal as a compartment-like syndrome. Future studies incorporating dynamic pressure monitoring, advanced neuroimaging, and biomechanical assessment during sitting and pelvic floor contraction may help definitively establish this pathophysiological model and improve both diagnosis and treatment strategies. The interpretation of these findings should also consider the methodological quality of the included studies. Although several studies consistently demonstrated similar anatomical and clinical observations, much of the available evidence originated from observational, retrospective, cadaveric, or imaging-based investigations with relatively small sample sizes. These methodological limitations reduce the certainty of causal inferences and reinforce that the proposed compartment-like mechanism should currently be regarded as a plausible pathophysiological model rather than definitive proof.

## Conclusions

PNE within Alcock’s canal demonstrates consistent evidence of chronic neurovascular compression occurring within a relatively confined fascial compartment, supporting a compartment-like pathophysiological mechanism. Clinical presentations, imaging findings, anatomical studies, and surgical observations collectively suggest that sustained pressure on the pudendal nerve can lead to ischemia, neural dysfunction, and chronic pelvic pain, similar to mechanisms observed in other compression neuropathies. Diagnostic approaches, including the Nantes criteria, MR neurography, and diagnostic nerve blocks, have improved recognition of the condition; however, no single modality currently serves as a definitive diagnostic gold standard. Management strategies range from conservative therapies to surgical decompression, with outcomes varying according to symptom duration, patient selection, and surgical technique. The most favorable results have generally been reported in patients undergoing timely and appropriately indicated decompression procedures. Although the available literature supports the concept of PNE as a compartment-like syndrome within Alcock’s canal, the overall evidence base remains limited by small sample sizes, heterogeneous study designs, and a lack of high-quality prospective research. Further standardized, multicenter studies are required to clarify the underlying pathophysiology, establish reliable diagnostic criteria, and optimize treatment pathways.
